# Pitch Enumeration: Failure to Subitize in Audition

**DOI:** 10.1371/journal.pone.0033661

**Published:** 2012-04-02

**Authors:** Neil M. McLachlan, David J. T. Marco, Sarah J. Wilson

**Affiliations:** School of Psychological Sciences, The University of Melbourne, Melbourne, Australia; Harvard University, United States of America

## Abstract

**Background:**

Subitizing involves recognition mechanisms that allow effortless enumeration of up to four visual objects, however despite ample resolution experimental data suggest that only one pitch can be reliably enumerated. This may be due to the grouping of tones according to harmonic relationships by recognition mechanisms prior to fine pitch processing. Poorer frequency resolution of auditory information available to recognition mechanisms may lead to unrelated tones being grouped, resulting in underestimation of pitch number.

**Methods, Results and Conclusion:**

We tested whether pitch enumeration is better for chords of full harmonic complex tones, where grouping errors are less likely, than for complexes with fewer and less accurately tuned harmonics. Chords of low familiarity were used to mitigate the possibility that participants would recognize the chord itself and simply recall the number of pitches. We found that accuracy of pitch enumeration was less than the visual system overall, and underestimation of pitch number increased for stimuli containing fewer harmonics. We conclude that harmonically related tones are first grouped at the poorer frequency resolution of the auditory nerve, leading to poor enumeration of more than one pitch.

## Introduction

Subitizing refers to the ability to rapidly estimate the number of distinct objects in the visual field. In speeded trials, estimation of numbers less than four appears rapid and effortless, whereas estimation of numbers greater than about 10 are intractable [1]–[4]. Visual number estimation is considered to involve both visual recognition of the array shape and serial counting mechanisms, and since visual recognition is faster it is the dominant mechanism for smaller arrays [4].

**Figure 1:**
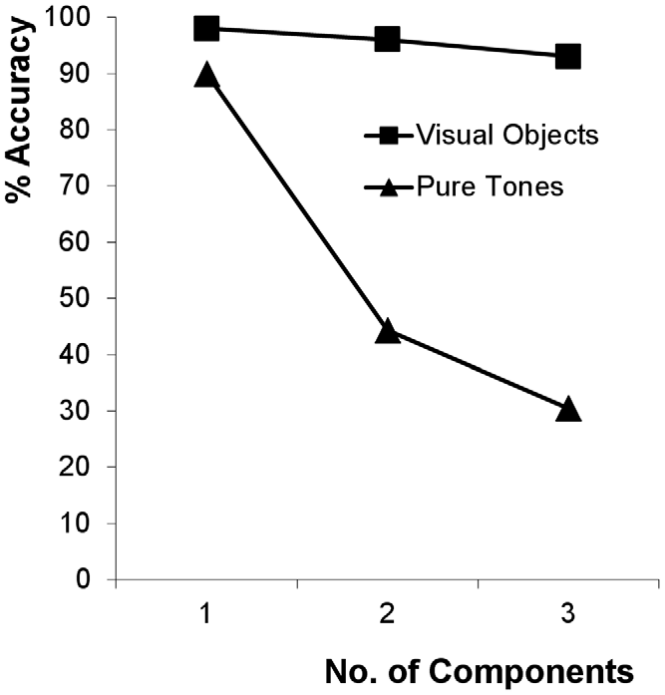
Comparison of number estimation accuracy for visual objects (redrawn from Vetter et al. [**4**], Figure 3A) and pure tones (experiment 5 in Thurlow and Rawlings [**6**]).

In the auditory domain people are able to match the pitch of pure tones to within 1% of frequency over a range of nearly 1000 Hz [5], suggesting that humans have ample frequency resolution to perceive multiple simultaneous pitches. An intriguing observation, however, is that estimates of the number of simultaneously presented pitches appear to be much less accurate than in visual subitizing (Figure 1). Thurlow and Rawlings [6] asked listeners to estimate the number of simultaneously presented pitches of pure tone stimuli, by reporting either one, two or three pitches. Their results showed at best 90% accuracy for 1-tone stimuli, but less than 50% accuracy for both 2- and 3-tone stimuli.

Most naturally occurring pitched sounds are harmonic complexes in which at least seven tones are arranged in integer multiples of the lowest, or fundamental frequency. In pitch perception the individual tones of harmonic complexes are grouped to produce the perception of a single pitch at the fundamental frequency [7], even when a tone at this frequency is absent [8]. The pitch strength of a harmonic complex is generally close to the average pitch strength of its individual harmonics [9]. Musicians have been shown to be better at grouping tones according to harmonic relationships than non-musicians [10], suggesting that this behavior can be learnt. McLachlan and Wilson proposed that this grouping involves recognition mechanisms that can be activated by partial representations of the stimulus, such as when the fundamental frequency is absent [11]. These mechanisms occur rapidly and likely prior to fine pitch processing of pitch height, and thus rely on the resolution of the cochlea. Since at any frequency, this resolution is at best about 15% [12], many concurrent tones could be incorrectly grouped. This suggests that some of the pure tones used by Thurlow and Rawlings [6] may have been incorrectly grouped into harmonic complexes, leading to systematic underestimation of the number of pitches in the stimuli. In support of this, Thurlow and Rawlings reported that the most frequent participant response was to underestimate the number of pitches in pure tone chords. Their stimuli consisted of synthesized tones at all possible 1-3 pitch combinations of tones at 750, 900, 1,200, 1,800, 2,400 and 3,000 Hz, so the resulting chords would be a wide cross-section of familiar, harmonically related tone intervals that may be mistaken for harmonic complex tones, and very unfamiliar, harmonically unrelated tone intervals.

**Figure 2:**
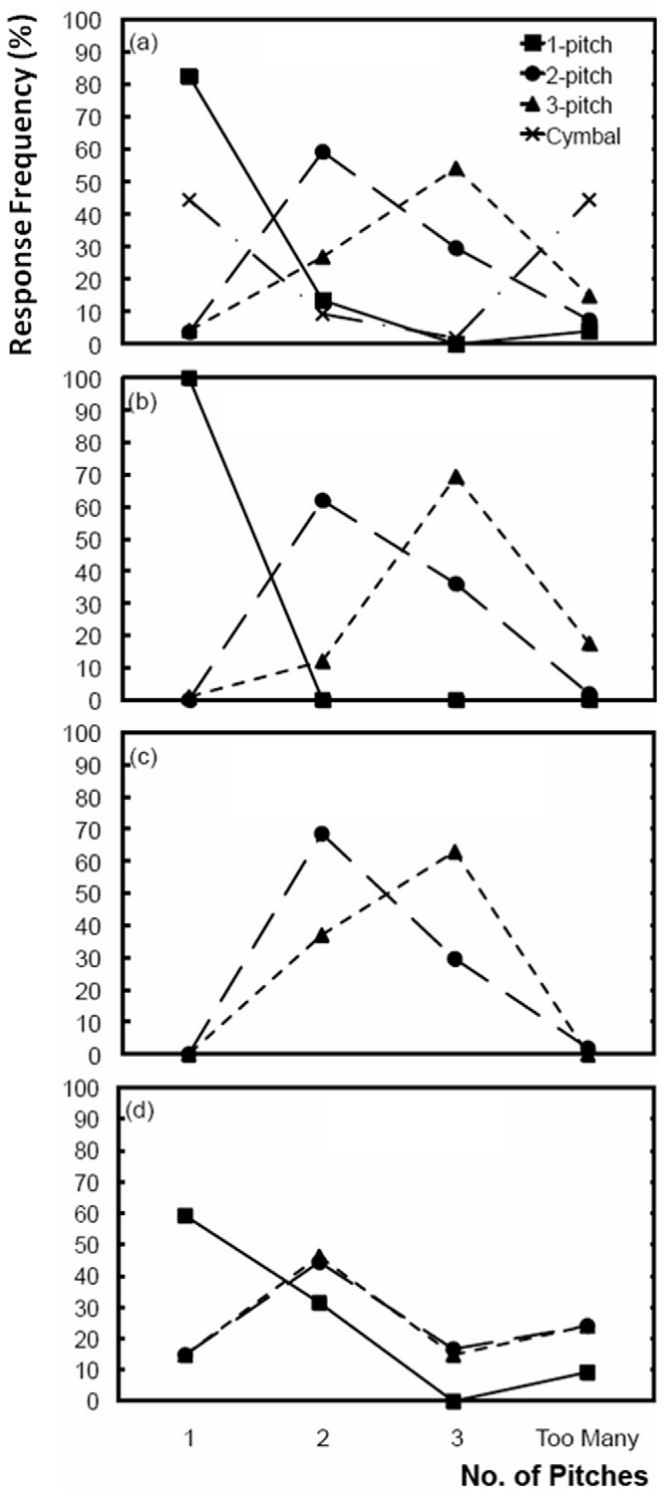
The percentage of correct responses for 1-pitch (squares), 2-pitch (circles), and 3-pitch (triangles) stimuli for A) all stimulus types compared to cymbals (crosses), B) harmonic complexes, C) reduced harmonic complexes, and D) bell sounds. Note: there was no 1-pitch data for the reduced harmonic complexes as pilot studies showed accuracy for these stimuli was the same as for full harmonic complexes.

In this study we investigated pitch enumeration accuracy for concurrent harmonic complex tones. More specifically, the enumeration accuracy of 1-pich harmonic complex tones and 2- and 3-pitch chords of harmonic complexes was compared to previous data reported by Thurlow and Rawlings [6] for pure tones. We also compared our data to that reported for visual enumeration of small numbers of objects [4].

We tested whether participants underestimated the number of pitches when the number of harmonics contributing to each pitch was reduced (reduced harmonic stimuli). This finding would suggest that participants incorrectly grouped the harmonics of two separate pitches, perceiving them as belonging to one pitch. We used synthesized chords of full and reduced harmonic complexes to test this in musical tunings that were all well resolved by the cochlea (with fundamental frequency differences greater than 15%). Chords of low familiarity were used to mitigate the possibility that participants would recognize the chord itself and simply recall the number of pitches. The frequency ratios of the fundamental frequencies of these unfamiliar chord tunings were sufficiently complex to ensure that they were not mistaken for a single harmonic complex with a virtual pitch at close to the common factor of the of the fundamental frequencies of each complex [13].

Finally the accuracy of pitch enumeration for natural instruments was assessed by including stimuli from real musical bells with tunings similar to the reduced harmonic stimuli but with some mistuned harmonics, varying harmonic amplitudes, and un-tuned higher frequency tones. These stimuli are representative of real tuned percussion instruments, and so provide an indication of the musical implications of these psychological mechanisms. As a comparison to the tuned stimuli we also used cymbal sounds, as these are inharmonic tonal stimuli that are unlikely to be grouped according to harmonic frequency relationships, and thus are not likely to produce consistent pitch number estimates.

## Results

Figure 2A shows the response frequencies for all stimuli with one, two or three pitches compared to the cymbal stimuli. Response curves for 1-, 2- and 3-pitch stimuli showed a peak in response frequency corresponding to the target number of pitches. For example, the highest percentage of responses (82.5%) for single-pitch stimuli fell on “1”, which was significantly greater than the number of responses across all other possible response categories χ^2^(1, N = 126) = 53.37, *p*<.01. Similarly, in the 2-pitch condition, participants reported hearing two pitches (62.4% of responses) significantly more often than all other categories combined χ^2^ (1, N = 216) = 7.41, *p*<.01. In contrast, greater uncertainty was evident in response to 3-pitch stimuli with no significant response consensus, despite the majority of responses occurring in the correct category (55.8% of responses).

To investigate the effects of using harmonic complex tones on the enumeration of 1-pitch tones and 2- and 3-pitch chords (Figure 2B and Table 1), a Wilcoxon signed-rank test was used to compare the median percentage of correct responses for each enumeration condition against the percentage correct values reported by Thurlow and Rawlings [6] for pure tones in their experiment 5. For the 1-pitch condition, a ceiling effect was observed (100% accuracy) suggesting that all participants were able to accurately enumerate a single pitched stimulus from a harmonic complex tone. Enumeration accuracy for harmonic complexes was significantly higher than the 90% accuracy reported by Thurlow and Rawlings for single pure tones (*T* = 4.24, *p*<.01). Similarly, responses for the 2- and 3-pitch conditions showed that chords of harmonic complex tones were enumerated more accurately than enumeration values for chords of pure tones reported by Thurlow and Rawlings, 2-pitch (*Mdn* = 66.8, *T* = 2.21, *p*<.05) and 3-pitch (*Mdn* = 66.6, *T* = 3.61, *p*<.01). Since all participants grouped tones tuned to the harmonic series as belonging to a single pitch, it is reasonable to expect that more than one harmonic complex would be heard as more than one pitch. However, similar to the data reported by Thurlow and Rawlings, enumeration accuracy for 2- and 3-pitch harmonic complex chords was still significantly below all values reported for visual enumeration of 2 and 3 objects reported by Vetter et al. [4] (>90% accuracy,), (*Mdn* = 66.7, *T* = 3.8, *p*<.01).

**Table 1:** Comparison of enumeration accuracy for harmonic complexes, pure tones and visual objects.

	Percentage of Correct Enumeration		
Number	Harmonic Complexes (this study)	Pure Tones (Thurlow and Rawlings, Exp. 5)	Visual Objects* (Vetter et al.)
1	100	90	97
2	66.8	44.3	95
3	66.6	30.5	92

* interpreted from Figure 3A, Vetter et al. (2008).

**Table 2:** Measured and ideal tuning ratios for the frequencies of the first 5 to 8 partials of the bells used in this study.

BELL DESCRIPTION		MEASURED AND (IDEAL) TUNING RATIOS							
Type 1^st^ partial Pitch ratios	Weight, height, diameter	Mode 1	Mode 2	Mode 3	Mode 4	Mode 5	Mode 6	Mode 7	Mode 8
**1-pitch 440** **Hz 1**	18 kg, 210 mm, 395 mm	**1**	**2.01** (2)	**2.98** (3)	**4.00** (4)	**5.08** (5)			
**2-pitch 294** **Hz 7/4**	132 kg, 520 mm, 570 mm	**1**	**1.79** (1.75)	**2.98** (3)	**3.53** (3.5)	**4.3** (4)	**5.5** (5.25)		
**3-pitch 294** **Hz 4/3+5/3**	340 kg, 800 mm, 1198 mm	**1**	**1.37** (1.33)	**1.71** (1.67)	**1.98** (2)	**2.87** (3)	**3.29** (3.33)	**3.93** (4)	**5.27** (5)

*Note: Ideal refers to the tuning of the finite element bell model, whereas measured refers to the overtone tuning in the sound of the physical prototype used in the study.*

A repeated measures analysis of variance (ANOVA) was conducted to investigate the effect of reducing the number of harmonics in 2- and 3-pitch chords on the accuracy of enumeration (Figure 2C vs. Figure 2B). Results from this analysis showed significant main effects for stimulus type, *F*(1,17) = 13.01, *p*<.01, and number of pitches, *F*(1,17) = 32.08, *p*<.01. An interaction was also observed between stimulus type and number of pitches, *F*(1,17) = 7.32, *p*<.05. To explore this interaction, two paired-samples t-tests were performed.

For the 2-pitch condition, results showed that there was no difference in the accuracy of enumeration between the stimulus types, *t*(17) = 0.85, *p*>.05. Whereas for the 3-pitch condition pitch number estimations were significantly less accurate for reduced harmonic complex chords (M = 2.63) versus harmonic complex chords (M = 3.04), *t*(17) = 3.96, *p*<.01. This is evidence for an underestimation of pitch number when 3-pitch chords are constructed using fewer harmonics.

A repeated measures ANOVA was also conducted to investigate the effect of additional mistuned harmonics present in real bell stimuli compared to the same set of 2- and 3-pitch reduced harmonic chords (Figure 2D vs. Figure 2C). Results from this analysis showed no significant main effects for stimulus type, *F*(1,17) = 0.00, *p*>.05, or number of pitches, *F*(1,17) = 2.52, *p*>.05. There was also no interaction between stimulus type and number of pitches, *F*(1,17) = 3.37, *p*>.05. Visual inspection of Figure 2D however reveals that despite no overall difference, there was greater enumeration uncertainty for real bell sounds, with participants frequently selecting responses “1” and “too many” for both 2- and 3-pitch chords. These response categories were not reported for reduced harmonic trials, suggesting that participants could more reliably identify that the reduced harmonic stimuli had more than one pitch but not more than three.

As expected, a significantly higher proportion of participants (89%) chose responses of “1” or “too many”, over “2” or “3” for the number of pitches in the cymbal stimuli χ^2^ (1, N = 54) = 32.67, *p*<.01. This suggests that the tones in cymbals were not being grouped and participants either attended to one particularly loud tone, or were aware of many concurrent tones and responded with “too many”.

## Discussion

This study showed that the accuracy of pitch enumeration for full harmonic complexes was greater than that previously reported for pure tones [6]. Despite reliably identifying the presence of multiple pitches in synthesized complexes, pitch number estimations for 2- and 3-pitch stimuli were substantially worse than 1-pitch stimuli. Taken together these data suggest that people can only reliably enumerate one pitch. This is in stark contrast to visual object number estimation in which object numbers of up to four can be rapidly estimated with very high accuracy [1]–[4]. The substantially different patterns of response between real bell and synthesized stimuli confirm that participants recognized the presence of harmonic frequency relationships in harmonic complex stimuli, but were confused by tuning errors and the presence of un-tuned tones in the bells.

In line with Thurlow and Rawlings [6], the expected number of pitches present in the stimulus was underestimated for 3-pitch reduced harmonic complex sounds. This supports the proposition that tones may be incorrectly grouped when the complete harmonic series for each pitch is not present in the stimulus. This was less likely to occur in 2-pitch chords as there were too many harmonics present for participants to mistake the stimulus for a 1-pitch complex. Similarly, DeWitt and Crowder [13] observed that people more often underestimated pitch number for 3-pitch chords of harmonic complexes when the combined series of tones approximated a single harmonic series due to a large number of coincident frequencies. However, this fusion effect was only observed for 3-pitch chords that contained octave (2∶1 frequency ratio) and perfect 5^th^ (3∶2 frequency ratio) intervals which were not used in this study.

The only existent model of pitch multiplicity [14] postulates that individual pitch salience are extracted from patterns of co-masking of spectral components, grouped according to harmonic relationships and then enumerated. This model cannot predict the very low accuracy of enumeration for 2-, and 3-pitch chords of pure tones reported by Thurlow and Rawlings [6], unless the harmonic grouping mechanism operates at a low frequency resolution. McLachlan and Wilson [11] proposed that sound recognition mechanisms correlate long-term memory templates for sound timbres with new auditory information as it emerges through time. The model proposes that spectral information present in onset patterns of auditory nerve responses is used to prime further processing of waveform information, leading to increasingly accurate pitch estimates over time [15]. This means that recognition of a set of harmonically tuned tones at the relatively low frequency resolution of the auditory nerve will lead to priming of pitch neurons near the fundamental frequency of the complex [9]. It is less clear how pitch height is perceived when more than one harmonic complex is present in the stimulus. A number of studies have investigated the ability of people to identify simultaneously presented vowels and match their pitches. Performance in simultaneous vowel identification improves with training [16], and as the frequency difference between the pitches increases above two semitones [17], which is less than the frequency resolution of the cochlea. These findings support the proposal that recognition mechanisms based on learnt chord templates at the frequency resolution of the cochlea may be involved in processing stimuli with multiple pitches.

To conclude the findings show that natural processing of musical chords does not usually result in multiple salient pitches that may be enumerated. This is likely due to the low resolution of grouping mechanisms that precede fine pitch processing. It is possible that templates could be used to recognize chords of known pitch relationships and subitize pitch number, as observed in a case study reported by Thurlow and Rawlings [6]. This hypothesis warrants further investigation, but was not investigated here as unfamiliar chords were used to increase the likelihood that recognition mechanisms would only operate on the harmonic relationships of tones associated with each pitch. Finally, pitch enumeration accuracy was reduced by the presence of higher frequency un-tuned tones in real percussion instrumental sounds. These instrumental sounds were very unfamiliar, which further supports the proposition that learnt templates are important in pitch processing.

## Materials and Methods

### Participants

All participants gave written informed consent and the experiments were carried out with the approval of the Human Research Ethics Committee of the University of Melbourne. Eighteen participants aged 18 to 31 years (6 males, mean age = 20.8) who reported receiving a minimum of six years musical training (M = 10.6, SD = 4.1) were recruited. We used musically trained participants to ensure that they were able to accurately find the pitch of a harmonic complex [9]. All participants underwent audiological and medical screening and were found to have normal hearing and no serious neurological or psychiatric conditions. Absolute pitch possessors were excluded by administering a pitch-naming task [18].

### Stimuli

Seven equal amplitude tones at harmonic frequencies were used to create 1-pitch harmonic complex tones. Chords were created by combining two or three harmonic complex tones using frequency intervals of the fundamental frequencies of each complex that are well resolved by the cochlea, but not frequently used in western music [19]. We created two 2-pitch chords comprising intervals of three semitones and six semitones, and two 3-pitch chords comprising intervals of five and eight semitones, and six and eight semitones. These chords were pitch shifted twice by one semitone to create twelve harmonic complex chord stimuli ranging from 294–784 Hz in their component pitches.

The 58 test stimuli comprised 16 synthesized full harmonic complex tones and chords, 30 synthesized reduced harmonic complex chords based on the ideal 2- and 3-pitch bell tunings shown in Table 1, 9 real bell sounds (Table 1), and three cymbal sounds. Fundamental frequencies varied between 262 and 784 Hz with a similar distribution for each stimulus type to control for possible variation of pitch strength with frequency. All synthesized stimuli were 500 ms duration with 10 ms linear fade on/fade off ramps. The first 500 ms of the bell and cymbal sounds were presented with 10 ms linear fade off ramps. Stimuli were normalized to 100% peak amplitude and presented at a fixed amplitude gain.

Bell sounds were recorded at a sample rate of 44,100 Hz and consisted of bells expected to produce 1-, 2-, and 3-pitches (Table 2). The 2- and 3-pitch bells were tuned so as to contain a fundamental frequency and a subset of the lower order harmonic frequencies of each intended pitch. The actual tuning was determined by the modal tunings possible for a real bell, as found by finite element modeling with shape optimization [20]. The 2-pitch bell was designed to produce a second pitch at a frequency ratio of 7/4, whereas the 3-pitch bell was designed to produce second and third pitches at frequency ratios 4/3 and 5/3 respectively. Reduced harmonic complex stimuli consisted of equal amplitude overtones at the frequency ratios listed for ideal tunings of the 2- and 3-pitch bells (Table 2). Ideal tunings were the targets for shape optimization of finite element models of the bells. Three cymbal sounds were used as stimuli likely to produce too many pitches due to their large number of inharmonic overtones.

### Experimental Procedure

Participants were tested individually in an anechoic chamber. Prior to the commencement of the experiment a short questionnaire assessing demographic information, relevant medical history, and music experience was administered. Sounds were presented monaurally through a loudspeaker (Alexis M1) placed in the middle of a table, one meter from the participant at head height. Each participant adjusted the amplifier gain to their preferred listening levels during pilot testing. Thereafter, stimuli were presented at the mean preferred listening level (SPL = 81±2 dB(A) fast response). Following the presentation of the sound, participants were asked to estimate how many pitches they heard. Responses for each stimulus were coded using pen and paper on a 4-point scale (“1”, “2”, “3” or “too many”). A response of zero was not included as pilot testing revealed that it was not selected even when participants were presented with complex cymbal sounds. Participants were instructed to report “too many” if they believed more than three pitches were present, or the sound comprised a complex array of frequencies from which no salient pitch was audible.

Three practice trials with feedback were conducted to ensure participants fully understood the task. The stimuli were then presented in three blocks of 26 trials and one block of 27 trials.

Participants were offered small breaks throughout the blocks of trials to minimize fatigue effects. Overall each experimental session lasted approximately 60 minutes.
